# Pharmacoacupuncture for Idiopathic Parkinson's Disease: A Systematic Review of Randomized Controlled Trials

**DOI:** 10.1155/2018/3671542

**Published:** 2018-06-25

**Authors:** Ki-Ho Cho, Tae-Hun Kim, Woo-Sang Jung, Sang-Kwan Moon, Chang-Nam Ko, Seung-Yeon Cho, Chan-Yong Jeon, Tae Young Choi, Myeong Soo Lee, Sang-Ho Lee, Eun Kyoung Chung, Seungwon Kwon

**Affiliations:** ^1^Department of Cardiology and Neurology, College of Korean Medicine, Kyung Hee University, Seoul, Republic of Korea; ^2^Korean Medicine Clinical Trial Center, Korean Medicine Hospital, Kyung Hee University, Seoul, Republic of Korea; ^3^Department of Korean Internal Medicine, College of Korean Medicine, Gachon University, Seongnam, Republic of Korea; ^4^Clinical Research Division, Korea Institute of Oriental Medicine, Daejeon, Republic of Korea; ^5^Gangdong Mokhuri Oriental Medical Hospital, Department of Internal Medicine, Seoul, Republic of Korea; ^6^Division of Clinical Pharmacy, Department of Pharmacy, College of Pharmacy, Kyung Hee University, Seoul, Republic of Korea

## Abstract

**Introduction:**

Pharmacoacupuncture is a new acupuncture treatment that stimulates acupuncture points by injecting herbal medicine into them. Recently, pharmacoacupuncture has been widely used in the treatment of idiopathic Parkinson's disease in traditional East Asian medicine. The purpose of this systematic review is to evaluate the efficacy and safety of pharmacoacupuncture in the treatment of idiopathic Parkinson's disease.

**Methods:**

The following electronic databases were searched for studies published in or before December 2016: Medline, Cochrane Central Register of Controlled Trials (CENTRAL), EMBASE, OASIS, and CNKI, without language restriction. The main outcome assessed was the total Unified Parkinson's Disease Rating Scale (UPDRS) score. The details of the pharmacoacupuncture intervention, such as the herbal medicine and acupuncture points used, were also investigated.

**Results:**

From 138 studies, 3 randomized controlled trials were included; the number of patients analyzed was 134. Most of the studies showed considerable methodological flaws. There was heterogeneity of the intervention type and treatment duration in the included studies. Therefore, we could not conduct a meta-analysis. In one study, adjunctive bee venom pharmacoacupuncture therapy significantly improved total UPDRS scores compared with conventional therapy alone. Another study, which used adjunctive Kakkonein pharmacoacupuncture, did not reveal significant improvement compared with conventional therapy alone. A third study reported that Mailuoning pharmacoacupuncture was able to significantly improve the modified Webster Symptom Score when compared with no treatment. Adverse events related to the pharmacoacupuncture were reported in only one case, itching caused by the bee venom.

**Conclusions:**

Our findings regarding the efficacy of pharmacoacupuncture as a therapy for idiopathic Parkinson's disease are currently inconclusive. Further large and rigorous clinical trials are needed.

## 1. Introduction

Idiopathic Parkinson's disease (IPD) is one of the most common chronic degenerative brain diseases. In the early stages, there are four major motor symptoms: bradykinesia, resting tremor, rigidity, and postural instability [1]. Thereafter, there are various nonmotor symptoms, such as neuropsychiatric symptoms, with chronic progression [1]. The hallmarks of IPD are progressive reduction in the number of dopaminergic neurons in the substantia nigra and continuous reduction of blood dopamine levels. The typical therapy for IPD with this pathology is pharmacotherapy, which takes the form of dopamine-elevating drugs, such as levodopa and dopamine agonists. Although these pharmacologic treatments initially show good efficacy, long-term levodopa therapy can cause on-off, peak-dose dyskinesia, and impulsive disorders as side effects [2]. Surgical procedures, such as deep brain stimulation (DBS), have also been attempted. However, DBS has potential neuropsychiatric side effects, such as apathy, cognitive dysfunction, and personality disorder [3], and carries additional risks, such as cerebral hemorrhage during surgery and postoperative brain edema [4]. Therefore, as the duration of treatment increases, most patients tend to seek alternative therapies. One study reported that 25.6–76% of patients were using complementary and alternative therapies [5].

In East Asian countries, besides typical alternative medical treatment, such as acupuncture and herbal medicine, a new form of acupuncture combining acupuncture with herbal medicine, called pharmacoacupuncture, [6] is being utilized in patients with IPD. Similar to traditional acupuncture, pharmacoacupuncture stimulates acupuncture points. In contrast to traditional acupuncture, pharmacoacupuncture injects herbal medicine extract with neuroprotective, antioxidant, and microcirculatory effects into acupuncture points, which are known to be beneficial to patients with IPD. It is believed that a synergistic effect may be achieved through the simultaneous use of herbal medicine and acupuncture; this technique is widely used in patients with IPD in East Asian countries, such as Korea. However, there has been no systemic review or meta-analysis to assess the efficacy of pharmacoacupuncture for IPD. The objectives of this review were to assess the clinical evidence on the efficacy and safety of pharmacoacupuncture in patients with IPD compared with control group (conventional therapy alone or no treatment) using randomized controlled trials (RCTs).

## 2. Methods

### 2.1. Search Strategy and Selection Criteria

We followed the methods of Lee et al. (2016) [7]. To evaluate the efficacy of pharmacoacupuncture (acupuncture point injection) in IPD, two independent authors (Seungwon Kwon and Tae-Hun Kim) searched for RCTs published in the following databases prior to December 2016: Medline, EMBASE, and the Cochrane Central Register of Controlled Trials (CENTRAL), which are English databases; the Chinese National Knowledge Infrastructure (CNKI; http://www.cnki.net), which is a Chinese database; and the Oriental Medicine Advanced Searching Integrated System (OASIS; https://oasis.kiom.re.kr), which is a Korean database. There were no language restrictions in this review. Each search strategy was modified according to the characteristics of the individual databases; however, primary search terms were “Parkinson disease” and “Acupuncture point injection.” Details of the employed search strategies are described in Supplementary Material (available here).

### 2.2. Eligibility Criteria

Eligible participants were defined as patients with IPD. Parkinson-plus syndromes and secondary Parkinsonism caused by drugs or toxins were exclusion criteria. Qualified clinical diagnosis methods, including the Queens Square clinical diagnostic criteria and the American Academy of Neurology practice parameters, were used for the diagnosis of IPD. There were no restrictions based on gender, ethnicity, symptom severity, disease duration, and clinical setting. Only the acupuncture point injection-type of pharmacoacupuncture intervention was included in this study. The intravenous injection-type of pharmacoacupuncture intervention was excluded. In addition, we excluded studies that combined pharmacoacupuncture with drug therapies other than conventional therapies for IPD. The application of pharmacoacupuncture was defined as a single treatment or combined treatment with conventional treatments, including anti-Parkinsonian medication or DBS. Control groups varied, with some receiving no treatment and others receiving only conventional treatments. We excluded studies involving other forms of pharmacoacupuncture, including manual acupuncture, electroacupuncture, and herbal medicine administered orally. We also excluded conventional interventions combined with alternative therapies such as herbal medicine, various other forms of acupuncture, or moxibustion.

### 2.3. Outcome Assessment

The primary outcome assessed was Unified Parkinson's Disease Rating Scale (UPDRS) scores. Secondary outcomes were total effective rate (TER) and modified Webster Symptom Scores. TER was defined as the percent of patients who showed improvement of their symptoms during treatment.

### 2.4. Data Extraction

Data extraction was assessed by two researchers (Seungwon Kwon and Ki-Ho Cho) and an arbiter (Tae-Hun Kim) made the final decision if there was disagreement between the researchers. Data recorded in a predefined extraction form included treatment protocol (intervention of pharmacoacupuncture group or control group), numbers of patients, country of publication, Parkinson's disease duration (years), outcome, and adverse events. Pharmacoacupuncture treatment details, such as treatment duration and frequency, acupuncture points used, and concomitant interventions, were extracted. If any of the above data was unclear, efforts were made to contact the authors of the study.

### 2.5. Assessment of Risk of Bias

Risk of bias (ROB) assessment was conducted by two independent authors (Seungwon Kwon and Ki-Ho Cho). In the event of a disagreement while extracting data or assessing ROB, the third author (Tae-Hun Kim) resolved the discrepancy. The domains of ROB assessment are random sequence generation, allocation concealment, participant and personnel blinding, outcome assessment blinding, incomplete data, selective reporting, and other biases according to the Cochrane Handbook for Systematic Reviews [8]. Each domain was graded as high, low, or unclear.

## 3. Results

### 3.1. Description of the Included Studies

**Figure 1:**
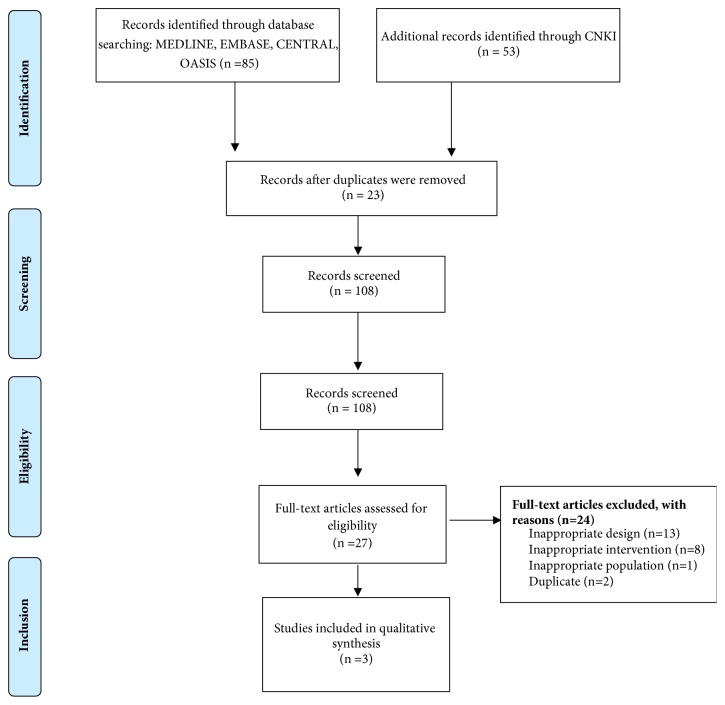
Study flowchart.

One hundred and thirty-eight studies were identified. One hundred and eight studies were assessed for eligibility after screening and removing duplications (Figure 1). Of these, 18 were excluded due to inappropriate intervention (not related to pharmacoacupuncture), population (not related to IPD), or study design (*in vivo* or* in vitro* studies and review articles). Three studies were ultimately chosen for this review. One RCT was conducted in South Korea [9], and two were conducted in China [10, 11]. One study was published in English [9], and the others [10, 11] were published in Chinese. The total number of patients analyzed in the review was 134 (pharmacoacupuncture group, 76; control group, 58). A summary of the included articles is presented in Table 1.

### 3.2. Details of the Included Intervention and Outcome Assessment

There were two intervention and control therapy types: (1) pharmacoacupuncture with conventional therapy versus conventional therapy alone [9, 10] and (2) pharmacoacupuncture versus no treatment [11]. In one trial where bee venom pharmacoacupuncture on GB20, LI11, GB34, ST36, and LR3 was used [9], severity of IPD symptoms was evaluated using UPDRS score. Patients with median IPD duration of 5 years were included. TER was used to evaluate the effect of Kakkonein pharmacoacupuncture on IPD symptoms in another RCT [10]. Kakkonein injection on bilateral GB20 was administered to patients with IPD with duration of 5.8 ± 2.5 years. Modified Webster Symptoms Score was used in remaining RCT [11]. In this RCT, Mailuoning injection was administered to bilateral ST36 and GB34. IPD duration [11] was 6.3 ± 2.0 years in the pharmacoacupuncture group and 6.7 ± 1.8 in the control group.

### 3.3. Risk of Bias in the Included Studies

**Figure 2:**
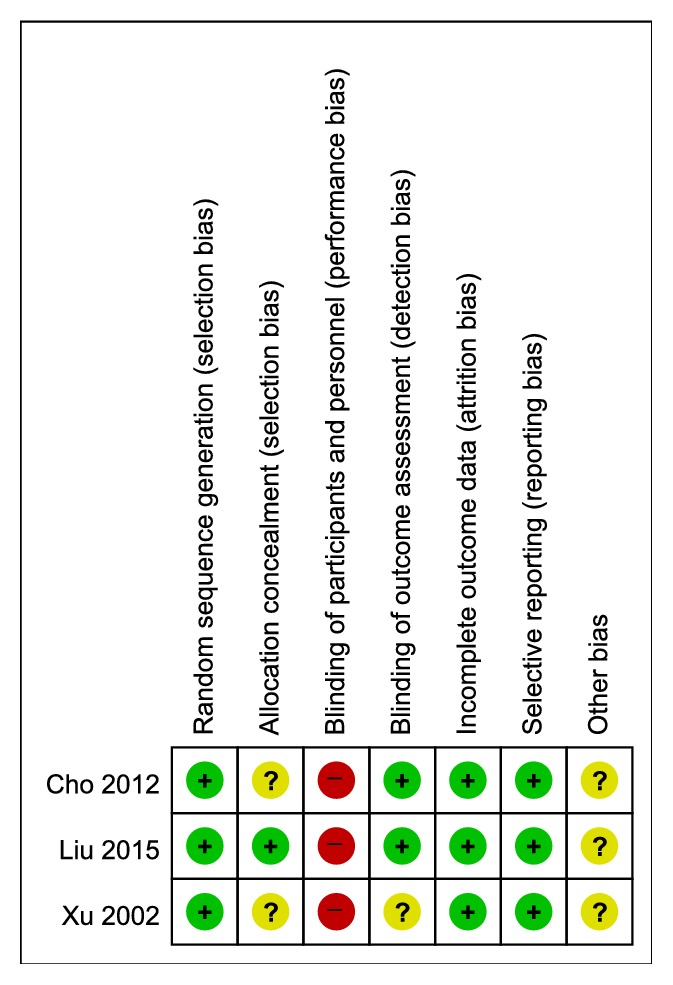
Risk of bias assessment in the included studies.

The overall risk of bias was not low. In particular, the bias related to participant blinding was relatively high. A summary of the risk of bias is shown in Figure 2.

#### 3.3.1. Random Sequence Generation

All three studies stated their use of a random number table [9–11].

#### 3.3.2. Allocation Concealment

Only one study reported that group allocation was done by an independent researcher [10]. The other two studies did not report their method of allocation concealment. The ROB for these studies was graded as unclear.

#### 3.3.3. Blinding of Participants and Personnel

None of the studies used a placebo pharmacoacupuncture device in order to blind participants [9–11]. Therefore, all these studies were graded as having high ROB.

#### 3.3.4. Blinding of Outcome Assessment

Two studies blinded the assessor [9, 10]. The other study [11] was graded as unclear because its description of assessor blinding was unclear.

#### 3.3.5. Incomplete Outcome Data

In one study [9], nine patients dropped out. Eight patients dropped out in another study [10]. However, the difference in dropout numbers between the two groups was not large, and the causes were similar; therefore these studies were graded as having low ROB. In another study [11], there were no drop-outs. This study was graded as having low ROB.

#### 3.3.6. Selective Reporting

We could not find published protocols for the included studies, but all of the variables mentioned in Methods were reported in Results. Therefore, all studies were graded as low ROB.

#### 3.3.7. Other Biases

There were no clues with which to evaluate other biases, so all studies were graded as unclear.

### 3.4. Outcome

None of the studies could be pooled into a quantitative analysis due to clinical heterogeneity in the intervention types and outcome assessments.

#### 3.4.1. Bee Venom Pharmacoacupuncture

Cho et al. [9] reported that 8 weeks of bee venom pharmacoacupuncture treatment in combination with anti-Parkinsonian medication for patients with IPD improved total UPDRS score (mean (lower quartile, upper quartile); 24.0 (17.5, 35.0) in the bee venom group, 38.0 (17.5, 53.5) in the control group, *p* < 0.05) (Table 1).

#### 3.4.2. Kakkonein Pharmacoacupuncture

Liu et al. [10] reported that TER of 8 weeks of Kakkonein pharmacoacupuncture treatment in combination with Madopa—compared to Madopa alone—was not significantly different (risk ratio (RR) 1.06, 95% confidence interval (CI) [0.90, 1.26]) (Table 1).

#### 3.4.3. Mailuoning Pharmacoacupuncture

Xu et al. [11] reported that 8 weeks of Mailuoning pharmacoacupuncture treatment—compared to no treatment—significantly improved total modified Webster Symptom Score (mean difference (MD) −6.94, 95% CI [−10.86, −3.02]) (Table 1).

### 3.5. Adverse Events

Only the RCT [9] using bee venom injection described adverse events. Itching, which can be caused by bee venom injection, occurred in only 1 case in the bee venom group. No adverse events were mentioned in other trials [10, 11] (Table 1).

## 4. Discussion

### 4.1. Summary of Main Findings

We conducted a systematic review of the clinical studies on the use of pharmacoacupuncture for treatment of IPD. Among the 138 studies identified through systematic searching, a total of three studies were included in this review, incorporating a total of 134 patients with IPD. Although every study showed high ROB in blinding of participants, most domains had either low or unclear ROB. Due to the heterogeneity of both the outcome assessments and the content of the pharmacoacupuncture injections used in the studies, we could not conduct an effective meta-analysis. Therefore, we do not make a definitive conclusion about the effect of pharmacoacupuncture on IPD. From the analyzed studies, we see that adjunctive administration of bee venom pharmacoacupuncture significantly improves the total UPDRS score of patients with IPD when compared with conventional therapy alone [9], and adjunctive administration of Kakkonein pharmacoacupuncture tends to improve overall symptoms in patients with IPD; however, it was not significantly improved compared with conventional therapy alone [10]. Finally, Mailuoning pharmacoacupuncture was able to significantly improve IPD patients' modified Webster Symptoms Score compared to no treatment [11]. Adverse events related to pharmacoacupuncture were reported in only one case, where itching was caused by administration of bee venom [9].

### 4.2. Strengths and Limitations of This Review

To our knowledge, this systematic review of the efficacy of pharmacoacupuncture in IPD treatment is the first of its kind. For the purpose of including as many literatures as possible, we did not impose any language restrictions on the studies analyzed; this review includes trials from South Korea and China. However, there were some limitations to this study. Due to heterogeneous study designs, including different types of pharmacoacupuncture, treatment durations, and outcome variables, we could not assess the estimated effect of pharmacoacupuncture through meta-analysis. First, there were differences in the comparison subjects. In two studies [9, 10], conventional therapies were maintained and pharmacoacupuncture was used as an adjunctive therapy. In the other study [11], the effect of pharmacoacupuncture was compared with no treatment. Second, the types of extraction injected in each study were different. One study [9] used bee venom, the second [10] used Kakkonein, and the last study [11] used Mailuoning. Third, there was a difference in treatment duration. Both studies continued for 8 weeks [9, 10], while the remaining study continued for 15 days [11]. Finally, there was a difference in the outcome variables in included studies. UPDRS [9], TER [10], and modified Webster Symptoms Score [11] were used in three studies, respectively.

Furthermore, we could not give a definitive conclusion regarding the efficacy of pharmacoacupuncture due to a lack of studies that met our inclusion criteria and methodological flaws in the included studies. Among three studies [9–11] included in this review, no study applied placebo to the control group. Therefore, performance bias was high. Furthermore, in one study [11], it was unclear whether blinding of outcome assessors was available. These methodological flaws should be improved in the future studies.

### 4.3. Interpretation of the Findings and Implications for Further Study

Despite the development of both pharmacotherapy and surgical treatment (e.g., deep brain stimulation), there are still limitations to the treatment of most patients with IPD. Pharmacotherapy, specifically with levodopa, causes an on-off phenomenon or peak dose dyskinesia after 5 years of “honeymoon period,” which significantly reduces the quality of life of patients [2]. In addition, surgical treatment is not an option for many patients because the cost is relatively high and there is a danger of adverse events, such as cerebral hemorrhage, after the procedure [4]. Therefore, recently, the use of various traditional and complementary medical therapies for the treatment of IPD has become an attractive option [5]. The most common treatment methods are herbal medicine and acupuncture. Pharmacoacupuncture combines these two treatments to inject herbal medicine extract directly into acupuncture points [6].

In this study, three types of pharmacoacupuncture were applied. The first utilized purified bee venom extracted by applying electromagnetic waves to* Apis mellifera* [12], the second technique, Kakkonein pharmacoacupuncture, used Kakkonein contained in* Pueraria* root, and the third treatment, Mailuoning pharmacoacupuncture, used a medicine called Mailuoning, which consists of Scrophulariae Radix, Dendrobii Herba, Achyranthis Radix, Lonicerae Flos, and* Codonopsis pilosulae* Radix.

The substances used in these pharmacoacupuncture therapies have documented neuroprotective effects. The primary outcome measure of our review is UPDRS score. This outcome was only evaluated in the trial involving administration of bee venom. This study confirmed that adjunctive application of bee venom pharmacoacupuncture could significantly improve total UPDRS scores. The mechanisms of action of bee venom in IPD are believed to be as follows [13]: (i) mitigation of neuroinflammation and microglial activation, (ii) suppression of apoptosis in dopaminergic neurons, (iii) protective effect against glutamate-induced neurotoxicity, and (iv) recovery of normal brain neurochemistry. Consistent with the proposed mechanisms of pharmacoacupuncture, 1 RCT [9] and 1 prospective open-label clinical study [14] have been published. Recently, an RCT using bee venom injection has been published [15].

In the other two studies, secondary outcomes, TER and the modified Webster Symptoms Score, were used to assess treatment effect. Although no significant effect was observed in the study where Kakkonein was administered [10], Kakkonein, which is one of the isoflavones of* Puerarin*, has anticholinergic effect and is likely to be a significant drug for IPD [16]. In the RCT using Mailuoning injectable solution, a 15-day treatment resulted in significant improvement in the modified Webster Symptoms Score of patients with IPD [11]. Mailuoning injection is a commonly used injection for acute ischemic stroke and is known to have a beneficial effect on neurological deficit [17]. In experimental and clinical pharmacological studies [17], it has been shown to improve blood circulation, prevent ischemic injury, and protect heart and brain tissue. In this study, Mailuoning injections were used as pharmacoacupuncture.

In pharmacoacupuncture, not only the injected solution but also the acupuncture points used are important. In this review, we saw that GB34, GB20, and ST36 were the most used. GB34 was used in 2 RCTs, GB20 was used in 2 RCTs, and ST36 was used in 2 RCTs. Of these, the acupuncture point with the most evidence of efficacy in IPD is GB34. GB34 has been reported to activate the precentral gyrus and prefrontal cortex in patients with Parkinson's disease [18], and another study has shown activation of neural responses in the substantia nigra, caudate, thalamus, and putamen of Parkinson's disease patients [19]. GB34 is thought to be helpful in improving Parkinson's disease symptoms by increasing the activity of the brain area associated with the development of movement disorders in patients with Parkinson's disease. ST36 has also been shown to have potential use in Parkinson's disease. In a previous experimental study [20], electroacupuncture on ST36 and SP6 in a mouse model of Parkinson's disease showed protective effects in the nigrostriatal system via antioxidative and antiapoptotic effects. In addition, a review of the effects of acupuncture on the adult brain [21] suggested that stimulation of ST36 could cause neurogenesis in the adult brain, although this effect may be specific to healthy individuals, even though it may be targeted to healthy subjects. Consequently, ST36 has the potential to prevent brain degenerative changes due to its antioxidant, antiapoptotic, and neurogenesis effects in Parkinson's disease patients. Although GB20 is also one of the most widely used acupuncture points, we could not find any significant effect in this review. There is also a lack of previous studies to support the effects of GB20. Therefore, based on our analysis, future clinical studies should investigate the effects of bee venom or Mailuoning as an injection and GB34 and ST36 as acupuncture points.

In contrasted with the other two studies [9, 11], no statistically significant effect was observed in the Kakkonein injection study [10]. There are two assumed reasons. (i) Kakkonein is known to have an anticholinergic effect [16]. However, the anticholinergic agents are currently used only as an adjunct in the treatment of Parkinson's disease. Therefore, there is a possibility that the effect of Kakkonein was limited due to its pharmacological properties. (ii) Only one acupuncture point, GB20, was used in this study. On the other hand, two other studies [9, 11] used 2 to 5 acupuncture points that could affect motor symptoms of Parkinson's disease. Therefore, in contrast with procedures performed at clinical sites, where multiple acupuncture points are used at once, the effect in this study might be limited.

### 4.4. Conclusion

In this review, we found few clinical studies on the effects of pharmacoacupuncture in Parkinson's disease, and meta-analysis could not be performed due to the heterogeneity of the interventions used in these studies. Therefore, we cannot make a concrete conclusion about the effect of pharmacoacupuncture on Parkinson's disease. However, from this review, we can derive recommendations for the types of injections and acupuncture points that could be used in future clinical studies. Future studies should include larger clinical trials with unified injectable solutions and unified acupuncture points to prevent intervention heterogeneity. In addition, as noted in this review, consideration of blinding is also important to improve ROB.

## Figures and Tables

**Table 1:** Summary of included randomized controlled studies.

First author, year	Total numbers of patients: pharmacoacupuncture group/control group	Groups		Main outcomes (Pharmacoacupuncture/control)	Adverse event
		Pharmacoacupuncture	Control group		Types of AEs in pharmacoacupuncture group (*n*=)/control group (*n*=)
		PD history (yrs)	PD history (yrs)		
		Protocol	Protocol		
Cho, 2012	22: 13/9	5.0 (median)	5.0 (median)	After 8 weeks: Total UPDRS 24.0 (median, 17.5, 35.0)/38.0 (17.5, 53.5)	1 (*n* = 18) Itching in 1 case/0 (*n* = 14)
		Bee venom (twice a week for 8 weeks (16 total sessions)) on bilateral GB20, LI11, GB34, ST36, LR3	No treatment		
		+ Antiparkinsonian medication	+ Antiparkinsonian medication	*p* < 0.05 calculated by Tukey's HSD post hoc test	

Liu, 2015	79: 40/39	5.8 ± 2.5	6.0 ± 3.1	After 8 weeks: TER (%) 90.0/84.6 -RR 1.06, 95% CI [0.90, 1.26]	No report
		Kakkonein (3 times a week for 8 weeks (24 total sessions)) on bilateral GB20 + Madopar	No treatment		
			+ Madopar		

Xu, 2002	33: 23/10	6.3 ± 2.0 Mailuoning (7 times a week for 15 days (15 total sessions)) on bilateral ST36, GB34	6.7 ± 1.8 No treatment	After 15 days: Modified Webster Symptom Scores 13.26 ± 4.00/20.20 ± 5.75 -MD −6.94, 95% CI [−10.86, −3.02]	No report

PD: Parkinson's disease; AEs: adverse events; UPDRS: Unified Parkinson's Disease Rating Scale; TER: total effective rate; RR: risk ratio; CI: confidence interval; MD: mean difference.

## Abbreviations

CENTRAL: Cochrane Central Register of Controlled Trials
CNKI: Chinese National Knowledge Infrastructure
DBS: Deep brain stimulation
IPD: Idiopathic Parkinson's disease
OASIS: Oriental Medicine Advanced Searching Integrated System
RCTs: Randomized controlled trials
ROB: Risk of bias
TER: Total effective rate
UPDRS: Unified Parkinson's Disease Rating Scale.

## Supplementary Materials

Supplementary Materials Search strategies in each database.
