# Supplementary material for: Pharmacoacupuncture for Idiopathic Parkinson's Disease: A Systematic Review of Randomized Controlled Trials
Source: Evid Based Complement Alternat Med. 2018 Jun 25;2018:3671542. doi: 10.1155/2018/3671542 (PMC6036809; doi:10.1155/2018/3671542)
Supplement: Supplementary Materials — Search strategies in each database. [file 3671542.f1.docx]

**Supplementary Material. Search Strategies in each database**

**MEDLINE**

Search (((((("acupuncture point injection"[tiab] or "acupoint injcetion"[tiab] or acup*[tiab]) and herb*[tiab])) OR ("Bee Venom*"[tiab] or apitoxin[tiab] or apitherapy[tiab] or "bee venom* acupuncture"[tiab] or "bee venom* therapy"[tiab] or "bee sting* therapy"[tiab] or "bee sting*"[tiab] or "herbal injection"[tiab])) OR "Bee Venoms"[Mesh])) AND (("Parkinson Disease"[Mesh]) OR ("Parkinson Disease"[tiab] OR "Parkinson*"[tiab]))

**CENTRAL**

| #1 | Parkinson Disease |
| --- | --- |
| #2 | Parkinson*:ti,ab,kw |
| #3 | #1 or #2 |
| #4 | Bee Venoms |
| #5 | bee venom* acupuncture:ti,ab,kw |
| #6 | bee venom* therapy:ti,ab,kw |
| #7 | "bee sting* therapy":ti,ab,kw |
| #8 | "bee venom" or "bee venom*" or apitoxin or apitherapy:ti,ab,kw |
| #9 | ("acupuncture point injection" or "acupoint injcetion" or acup*) and herb*:ti,ab,kw |
| #10 | "bee sting" or "bee sting*":ti,ab,kw |
| #11 | "herbal injection":ti,ab,kw |
| #12 | #4 or #5 or #6 or #7 or #8 or #9 or #10 or #11 |
| #13 | #3 and #12 |

**EMBASE**

('parkinson disease'/exp OR 'parkinson*':ab,ti) AND ('bee venom'/exp OR ('bee venom*':ab,ti OR 'pharmacopuncture*':ab,ti OR 'bee venom* acupuncture':ab,ti OR 'bee venom* therapy':ab,ti OR 'bee sting* therapy':ab,ti OR 'bee sting*':ab,ti OR apitoxin OR apitherapy) OR ('acupuncture point injection':ab,ti OR 'acupoint injcetion':ab,ti OR acup*:ab,ti AND herb*:ab,ti) OR 'herbal injection*':ab,ti)

**OASIS**

파킨슨 and 약침

**CNKI**

| #1 | 帕金森病 |
| --- | --- |
| #2 | 帕金森氏病 |
| #3 | 震颤麻痹 |
| #4 | 颤病 |
| #5 | 颤证 |
| #6 | 颤震 |
| #7 | 颤拘病 |
| #8 | 振掉 |
| #9 | 拘病 |
| #10 | Parkinson Disease |
| #11 | OR/ #1-#10 |
| #12 | 穴位注射 |
| #13 | 药针 |
| #14 | 蜂针 |
| #15 | Point injection |
| #16 | OR/ #12-#15 |
| #17 | 11 AND #16 |
